# Clinicopathological significance and prognostic values of claudin18.2 expression in solid tumors: a systematic review and meta-analysis

**DOI:** 10.3389/fonc.2024.1453906

**Published:** 2024-11-20

**Authors:** Gyerim Park, Se Jun Park, Younghoon Kim

**Affiliations:** ^1^ Department of Hospital Pathology, Seoul St. Mary’s Hospital, College of Medicine, The Catholic University of Korea, Seoul, Republic of Korea; ^2^ Division of Medical Oncology, Department of Internal Medicine, Seoul St. Mary’s Hospital, College of Medicine, The Catholic University of Korea, Seoul, Republic of Korea

**Keywords:** claudin18.2, carcinoma, immunohistochemistry, clinical significance, meta-analysis

## Abstract

**Objective:**

Claudin18.2 has been established as a putative therapeutic target in human solid malignancies. The aim of this study is to determine claudin18.2 expression as a clinicopathological and prognostic factor in human solid tumors through a systematic review and meta-analysis. Articles were systematically reviewed for studies that included the correlation between claudin18.2 expression and clinicopathological features and prognosis in solid tumors. Meta-analysis was conducted to estimate either odds ratio and 95% confidence intervals (CIs) of clinicopathological factors or hazard ratio and 95% CIs of survival outcomes for claudin18.2 expression in all available solid tumors.

**Results:**

21 studies including 5,331 patients were identified. Overall proportion of claudin18.2 positivity was 29.7%. Analyses of clinicopathological features demonstrated that claudin18.2 positivity correlated with male predominance, lower T stage, more frequent MUC5AC positivity when all primary tumors included. In subgroup analysis, gastric cancer showed significant correlation between high claudin18.2 expression and frequent EBV infection, male predominance and lower T stage. In lung cancer, claudin18.2 expression was associated with favorable overall survival. However, analyses of survival outcomes in all solid tumors showed that claudin18.2 expression was not associated with overall survival and pooled disease-free survival, tumor-specific survival, progression-free survival and relapse-free survival.

**Conclusions:**

Our study emphasizes evaluation of claudin18.2 expression as a potential prognostic factor in lung adenocarcinoma and further exploration in other solid tumors as well.

**Systematic review registration:**

https://www.crd.york.ac.uk/prospero/, identifier CRD42023468651.

## 1 Introduction

Claudins are a family of proteins that play a crucial role in cell-to-cell adhesion at tight junctions (1). Encoded by various claudin genes, these proteins exhibit diverse expression patterns in different normal tissues. While some claudins are widely distributed throughout the body, others are selectively expressed in specific organs. For example, claudin-1 is normally found in various organs, whereas claudin-5 is limited to the brain and pancreas, and claudin-7 is predominantly expressed in the kidney, lung, and prostate (2). Channel-forming claudins create channels between adjacent cells. Defects in channel-forming claudins can lead to metabolic disorders. For instance, claudin-2 knock-out results in decreased bile flow and gastrointestinal issues like diarrhea. Barrier-forming claudins contribute to maintaining tight junction integrity. Abnormal expression of barrier-forming claudins is associated with inflammation and carcinogenesis (3).

The claudin-18 gene expression results in two distinct isoforms, claudin18.1 and claudin18.2, differentiated by alternative splicing of their first exon (4). Claudin18.1 is expressed in the normal lung epithelium, whereas claudin18.2 in the normal gastric mucosa. Notably, claudin18.2 has been implicated in several carcinomas, making it a putative pathogenic factor, while claudin18.1 has been recognized to be linked to asthma and impaired alveologenesis (5, 6).

In the stomach, claudin18.2 acts as a barrier against protons. Loss of claudin 18.2 can lead to gastritis and it was reported that in mice models, knock-out of claudin18.2 in the stomach develops gastric cancer (3). Translocation involving the claudin18 gene contributes to gastric cancer tumorigenesis by promoting the loss of epithelial phenotype and inducing epithelial-mesenchymal transition (7). Other than gastric cancer, aberrant expression of claudin18.2 is also observed in other solid tumors, including pancreatobiliary, esophageal, colorectal, small bowel, and lung cancers (8–12). For example, claudin18.2 is expressed in 60–90% of pancreatic ductal adenocarcinoma (13).

Claudin18.2 targeted therapies in gastric cancer have emerged and several clinical trials and studies are actively on going. Moreover, claudin18.2’s involvement in solid tumor development highlights its potential as a therapeutic target in cancer research. However, to date there has never been a reported investigation of claudin18.2 expression in overall human solid tumors. Herein, we reviewed the expression of claudin18.2 across human solid malignancy. Furthermore, clinicopathological features and prognosis of claudin18.2 expressing solid tumors in overall organs were comprehensively investigated.

## 2 Materials and methods

### 2.1 Publication search strategy

This study followed the PRISMA guidelines for reporting systematic reviews and meta-analyses. We searched PubMed, Embase, Cochrane, and Web of Science databases for articles related to the topic up to October 14, 2023. The search strategy combined the following terms: (“claudin-18”, “claudin18”, OR “claudin18”), (“malignancy”, “malignant tumor”, “solid tumor”, “carcinoma”, OR “cancer”), AND (“prognosis”, “survival”, “prognostic”, OR “outcome”). We also checked the reference lists of relevant systematic reviews for additional studies. Two pathologists (GP and YK) independently screened the titles and abstracts of the retrieved articles and resolved any disagreements by consensus. This review was registered on the International Prospective Register of Systematic Reviews (registration number: CRD42023468651).

### 2.2 Inclusion and exclusion criteria

We included full-text articles published in English that met the following criteria: (1) they assessed claudin18.2 expression by immunohistochemistry (IHC) in human tumor samples obtained from surgery or biopsy; (2) they classified the samples into positive and negative (or high and low) groups based on the IHC staining intensity and/or percentage; and (3) they reported clinicopathological or survival outcomes associated with claudin18.2 expression. We excluded studies that were: (1) case reports, reviews, abstracts, or posters; (2) unable to provide dichotomous data on claudin18.2 expression; or (3) based on samples that received neoadjuvant chemoradiotherapy.

### 2.3 Data extraction and quality assessment

The following data were extracted from each article: authors’ name, year of publication, number of total patients, number of claudin18 positive patients, type of claudin18 IHC, definition of claudin18.2 positivity, primary site of cancer, clinicopathological data, and survival data. We performed a pooled analysis of the clinicopathological features that were common to at least three studies. Survival data were classified into overall survival (OS) and disease-free survival (DFS), tumor-specific survival (TSS), progression-free survival (PFS), or relapse–free survival (RFS). The hazard ratio (HR) and 95% confidence interval (CI) were obtained for each survival outcome. When the survival data were only presented as Kaplan-Meier curves without HR, we estimated them using Engauge Digitizer V9.8 and the spreadsheets provided by Tierney et al. (14) We assessed the quality of the studies using the Newcastle-Ottawa Scale and included studies with scores higher than six.

### 2.4 Statistical analysis

We analyzed the pooled OR and 95% CI for clinicopathological features and the pooled HR and 95% CI for OS and DFS/TSS/PFS/RFS. Subgroup analyses were conducted for different primary sites of cancer within each pooled analysis. Statistic heterogeneity was determined by Cochrane’s Q and *I* ^2^. When heterogeneity was observed (*p* < 0.05 or *I* ^2^ > 50%), a random effect model was used for the analysis instead of a fixed effect model. The risk of bias was assessed through a funnel plot. All of these statistical analyses were performed using Comprehensive Meta-Analysis Software (CMA; version 4; Englewood, NJ 07631 USA).

## 3 Results

### 3.1 Literature search and study characteristics

**Figure 1:**
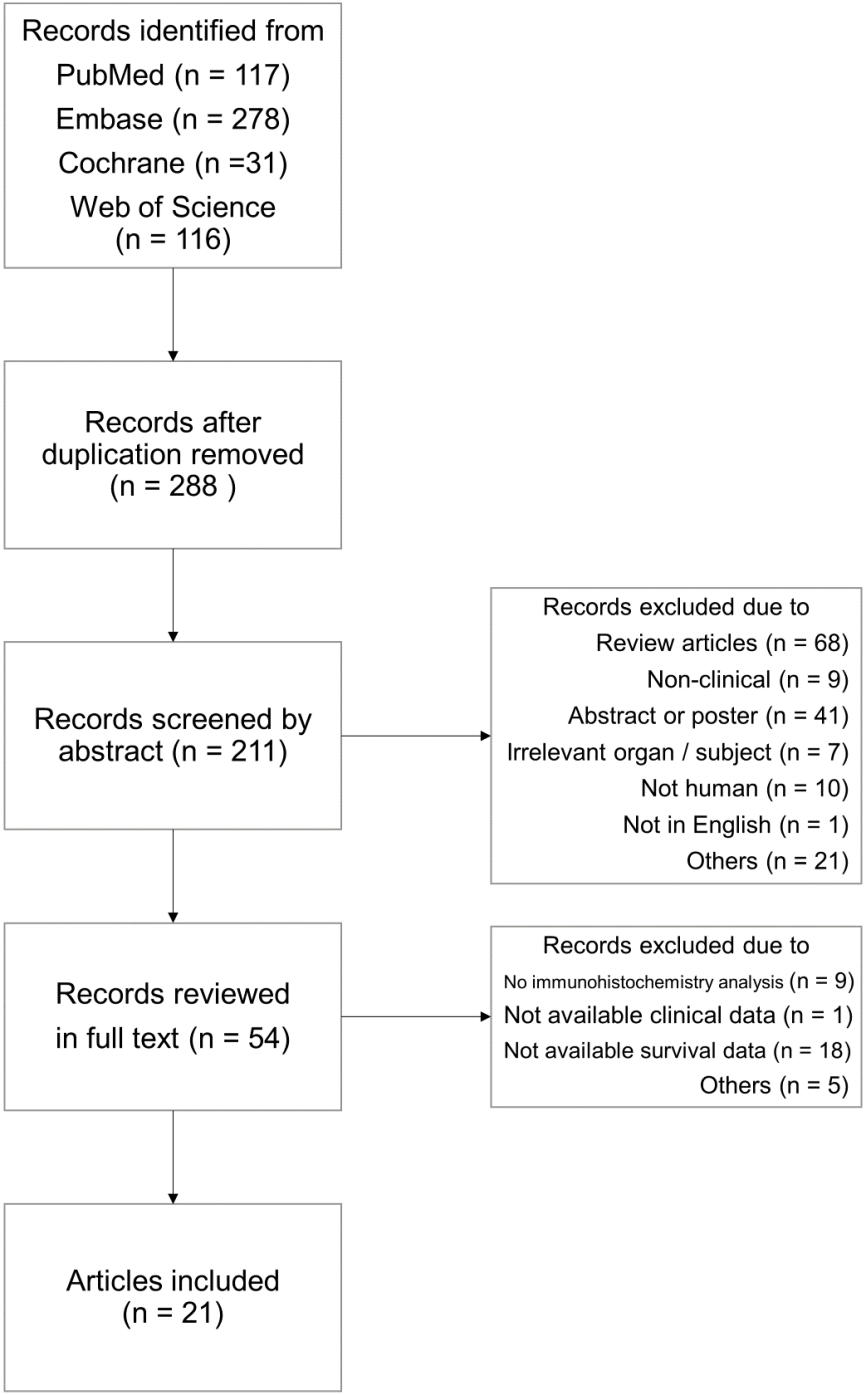
Flow chart illustrating the search and the study selection.

From databases, 542 records were retrieved with 254 duplicates. After examining the titles and abstract, the remaining 54 articles were reviewed in full texts. Finally, 21 articles were included as being relevant to the subject (**Figure 1**) (15–35). General characteristics of 21 studies were listed in **Table 1**. They were from eight different countries (Korea, China, Japan, Germany, Brazil, Finland, Italy, and United States) and published between 2006 and 2023. Targeted organs included the stomach, pancreas, biliary tract, small bowel, and lung. Intriguingly, all included studies were based on adenocarcinoma of these organs. Among them, stomach held a majority. A total of 5,331 patients were involved in our study, 1,585 (29.7%) patients expressing claudin18.2 positivity. By cancer types, the rates of claudin18.2 positivity were 39.3%, 54.5%, and 9.0% in gastric, pancreatic, and lung cancers, respectively. Each study established a different cut-off value between claudin18.2 positive and negative samples. Furthermore, there was diversity in IHC antibody used for claudin18.2 staining. The majority of studies used the products manufactured in Abcam (42.9%) or Invitrogen (19.0%). 18 studies reported OS and 7 studies reported DFS/TSS/PFS/RFS.

**Table 1:** Characteristics of included studies.

Author	Year	Country	Organ	No. of patients	Claudin18.2 + patients (%)	Availability	Source	Survival	Definition of overexpressed claudin18.2	NOS
Sanada, Y., et al. (15)	2006	Japan	Stomach	146	27 (18.5%)	KM	Univariate	OS	≥ 50%	8
Karanjawala, Z.E., et al. (16)	2008	United States	Pancreas	166	83 (50%)	Cox	Multivariate	OS	Strongly and diffusely labeled	7
Matsuda, M., et al. (17)	2010	Japan	Colorectum	569	21 (3.7%)	Cox	Multivariate	NA	≥ 10%	7
Merikallio, H., et al. (18)	2011	Finland	Lung	108	23 (21.3%)	KM	Univariate	OS	≥ 5% membrane bound	8
Shinozaki, A., et al. (19)	2011	Japan	Biliary tract	83	36 (43.4%)	KM	Univariate	OS, DFS	> 0%	8
Jun, K.H., et al. (20)	2014	Korea	Stomach	134	65 (48.5%)	Cox	Univariate	OS	≥ 50%	8
Baek, J.H., et al. (21)	2019	Korea	Stomach	367	108 (29.4%)	Cox	Multivariate	OS, DFS	≥ 51% with moderate to strong staining intensity	8
Dottermusch, M., et al. (22)	2019	Germany	Stomach	430	249 (57.9%)	KM	Univariate	TSS	visible membrane staining	8
Arnold, A., et al. (23)	2020	Germany	Stomach	381	65 (17.1%)	KM	Univariate	OS	Immunoreactive score (IRS) (1) > 8	7
Hong, JY., et al. (24)	2020	Korea	Stomach	81	12 (14.8%)	KM	Univariate	NA	> 5% with weak membrane staining	8
Lu, Y., et al. (25)	2020	China	Stomach	63	27 (42.9%)	KM	Univariate	OS	–	8
Xu, B., et al. (26)	2020	China	Stomach	105	68 (64.8%)	KM	Univariate	OS	Staining intensity ≥ 2+ in ≥ 40% of cells	7
Pereira, M.A., et al. (27)	2021	Brazil	Stomach	349	176 (50.4%)	Cox	Univariate	OS, DFS	≥ 33% membrane staining with ≥ 1+ intensity	7
Arpa, G., et al. (28)	2022	Italy	Small bowel	78	23 (29.5%)	KM	Univariate	OS	≥ 1% membrane staining with ≥ 1+ intensity	7
Liu, J., et al. (29)	2022	China	Lung	1079	84 (7.8%)	KM	Univariate	OS	Score (2) 2-9	7
Wang, X., et al. (30)	2022	China	Pancreas	80	39 (48.8%)	KM	Univariate	TSS	H-score (3) > 0	8
Xu, B., et al. (31)	2022	China	Stomach	44	14 (31.8%)	KM	Univariate	PFS	≥ 40% membrane staining with ≥ 2+ intensity	7
Jia K., et al. (32)	2022	China	Stomach	80	42 (52.5%)	KM	Univariate	OS	≥ 40% membrane staining with ≥ 2+ intensity	8
Park, S., et al. (33)	2023	Korea	Pancreas	123	79 (64.2%)	Cox	Univariate	OS, RFS	≥ 80% membrane staining with ≥ 2+ intensity	8
Tao, D., et al. (34)	2023	China	Stomach	414	99 (23.9%)	KM	Univariate	OS	IRS ≥ 8	7
Wang, C., et al. (35)	2023	China	Stomach	451	245 (54.3%)	Cox	Multivariate	OS	H-score ≥ 1	8

KM, Kaplan-Meir curve; NOS, Newcastle-Ottawa Scale.

(1) IRS = percentage of stained tumor cells (0 = 0%, 1-1-25%, 2-26-50%, 3 = 51-75%, 4 = 76-100%) x staining intensity (0-3).

(2) Score = intensity {negative (0), weak (1), moderate (2), and strong (3)} x fraction of tumor cells stained {< 1% (0), 1-9% (1), 10-50% (2), > 50% (3)}.

(3) H-score = {0 × (percentage of immunonegative cells)} + {1 × (percentage of weakly stained cells)} + {2 × (percentage of intermediately stained cells)} + {3 × (percentage of strongly stained cells)}.

### 3.2 Pooled analysis and between claudin18.2 expression and clinicopathological features

Pooled analysis for correlation between claudin18.2 and clinicopathological features is described in **Table 2**. The claudin18.2 positive group had a higher proportion of male patients (OR, 1.578; 95% CI, 1.056 - 2.358; *p* = 0.026), was associated with lower T stage (OR, 0.822; 95% CI, 0.692 - 0.976; *p* = 0.026), and showed more frequent positivity for MUC5AC (OR, 3.899; 95% CI, 1.228 - 12.382; *p* = 0.021) when all studies were considered. In more detailed analysis, claudin18.2 expression showed a decreasing trend across pT1, pT2, and pT3 stages. However, this trend was not observed in the pT4 stage, where claudin18.2 expression did not continue to decline (**Supplementary Figure S1**). Epstein-Barr virus (EBV) status, which was evaluated only in gastric cancer, revealed that high claudin18.2 was correlated with frequent EBV infection (OR, 3.962; 95% CI, 2.083 - 7.534; *p* < 0.001). In subgroup analysis (**Table 3**), studies with gastric cancer showed significant correlation between high claudin18.2 expression and male predominance (OR, 1.955; 95% CI, 1.142 – 3.347; *p* = 0.015) and lower T stage (OR, 0.828; 95% CI, 0.687 – 0.998; *p* = 0.047). Similar to overall malignancies, claudin18.2 expression demonstrated a decreasing trend from pT1 to pT3 stages, but this trend was not observed in the pT4 stage (**Supplementary Figure S1**).

**Table 2:** Pooled analysis between claudin 18 expression and clinical features.

Clinical features	No. of Studies	M-H OR	95% CI	*p*-value
Age	10	1.048	0.853 - 1.284	0.660
Sex (male/female)	16	1.580	1.053 - 2.370	0.027*
Size	3	0.892	0.641 - 1.242	0.499
Differentiation	11	0.982	0.664 - 1.452	0.927
pT	12	0.809	0.680 - 0.963	0.017*
pN	14	0.945	0.756 - 1.180	0.616
pM	9	1.101	0.980 - 1.461	0.504
Stage	14	0.921	0.808 - 1.119	0.542
Lymphatic invasion	5	0.904	0.720 - 1.135	0.386
Vascular invasion	8	0.818	0.632 - 1.058	0.126
Perineural invasion	7	1.212	0.647 - 2.272	0.548
Mismatch repair	5	0.746	0.503 - 1.106	0.145
MUC2	3	0.929	0.337 - 2.559	0.887
MUC5AC	4	3.962	1.077 - 14.572	0.038*
MUC6	4	2.038	0.678 - 6.127	0.205
EBV (stomach only)	4	3.962	2.083 - 7.534	< 0.001*
E-cadherin (stomach only)	3	1.143	0.811 - 1.611	0.445
HER2 (stomach only)	8	0.739	0.450 - 1.211	0.230

M-H OR, Mantel-Haenszel odds ratio; CI, confidence interval.

* p < 0.05.

**Table 3:** Subgroup analysis for clinical features.

Clinical features	Organ	Study	OR	95% CI	*p*-value
Age	Stomach	7	0.978	0.782 - 1.223	0.847
Sex (male/female)	Stomach	11	1.955	1.142 - 3.347	0.015*
	Pancreas	2	0.711	0.410 - 1.231	0.223
Differentiation	Stomach	7	1.096	0.705 - 1.702	0.685
	Pancreas	2	0.578	0.239 - 1.400	0.225
pT	Stomach	8	0.828	0.687 - 0.998	0.047*
	Pancreas	2	0.888	0.483 - 1.633	0.703
pN	Stomach	9	0.903	0.765 - 1.065	0.224
	Pancreas	2	1.002	0.530 - 1.897	0.994
pM	Stomach	6	0.975	0.721 - 1.320	0.872
Stage	Stomach	10	0.948	0.796 - 1.130	0.554
	Pancreas	2	1.001	0.464 - 2.159	0.998
Lymphatic invasion	Stomach	4	0.893	0.704 - 1.132	0.348
Vascular invasion	Stomach	5	0.845	0.636 - 1.124	0.247
	Pancreas	2	0.680	0.295 - 1.565	0.364
Perineural invasion	Stomach	3	0.611	0.272 - 1.373	0.233
	Pancreas	2	2.181	0.734 - 6.482	0.161
Mismatch repair deficiency/Microsatellite instability	Stomach	4	0.792	0.522 - 1.203	0.274

OR, odds ratio; CI, confidence interval.

* p < 0.05.

### 3.3 Pooled analysis between claudin18.2 expression and survival

**Figure 2:**
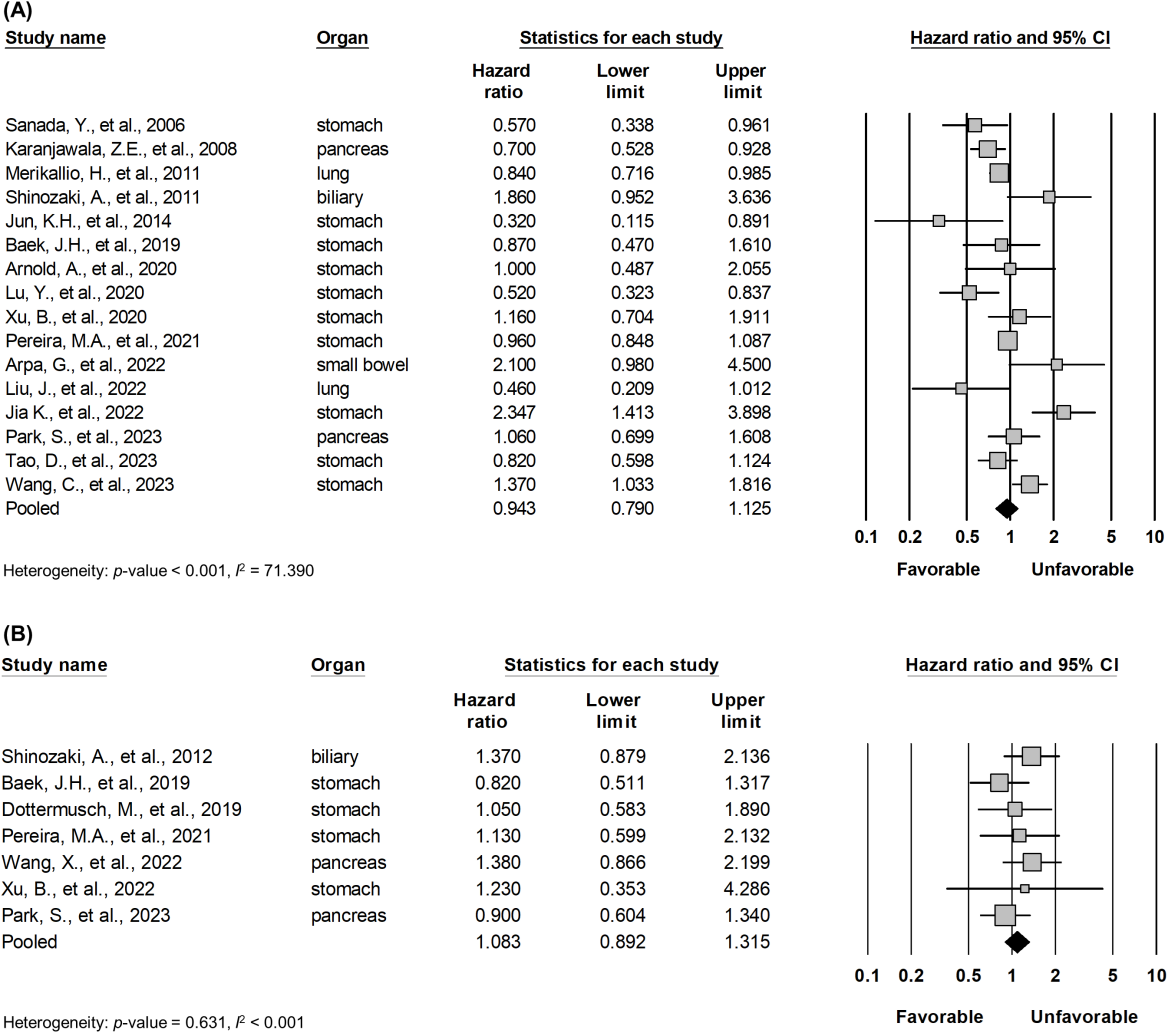
Forest plot describing the correlation between claudin18.2 expression and OS **(A)** and DFS/TSS/PFS/RFS **(B)**.

Pooled analysis between OS and DFS/TSS/PFS/RFS (**Figure 2**) in all solid tumors did not show significant correlation between claudin18.2 expression and survival (HR, 0.943; 95% CI, 0.790 - 1.125; *p* = 0.511; and HR, 1.083; 95% CI, 0.892 - 1.315; *p* = 0.422 for OS and DFS/TSS/PFS/RFS, respectively). However, in subgroup analysis (**Table 4**), studies with lung cancer retained favorable prognostic significance between claudin18.2 expression and OS (HR, 0.820; 95% CI, 0.702 – 0.959; *p* = 0.013). In contrast, such prognostic significance was not observed in cases of gastric and pancreatic cancer (HR, 0.931; 95% CI, 0.730 – 1.188; *p* = 0.567; and HR, 0.798; 95% CI, 0.631 – 1.007; *p* = 0.058, respectively).

**Table 4:** Subgroup analysis for survivals.

Survival	Organ	Study	HR	95% CI	*p*-value
OS	Stomach	10	0.931	0.730 - 1.188	0.567
	Pancreas	2	0.798	0.631 - 1.007	0.058
	Lung	2	0.820	0.702 - 0.959	0.013*
DFS/TSS/PFS/RFS	Stomach	4	0.971	0.713 - 1.323	0.853
	Pancreas	2	1.078	0.797 - 1.459	0.627

HR, hazard ratio; CI, confidence interval; OS, overall survival; DFS/TSS/PFS/RFS, disease-free survival/tumor-specific survival/progression-free survival/relapse-free survival.

* p < 0.05.

### 3.4 Publication bias

**Figure 3:**
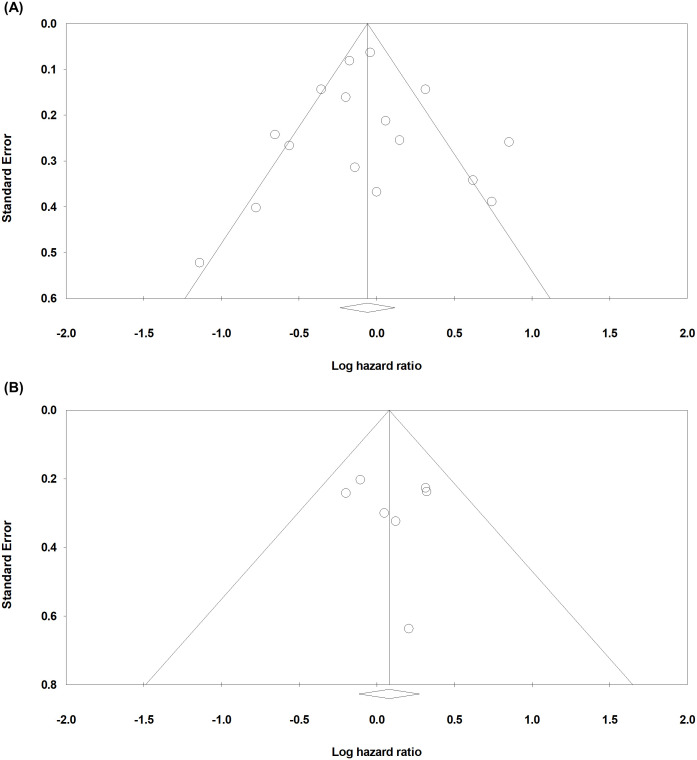
Funnel plots to evaluate publication bias in studies with OS **(A)** and DFS/TSS/PFS/RFS **(B)**.

Begg’s funnel plot was evaluated for the presence of potential publication bias in meta-analysis associated with survival (**Figure 3**). Egger’s test reported *p*-values of 0.459 for OS and 0.374 for DFS/TSS/PFS/RFS. These results demonstrated that publication bias is unlikely. Sensitivity test showed that omitting any single study did not result in significant correlation between claudin18.2 expression and survival (data not shown).

## 4 Discussion

Claudin18.2 is an emerging therapeutic target for advanced gastric or gastro-esophageal junction cancer (36). Zolbetuximab, a chimeric monoclonal antibody drug, induces the immune-mediated lysis of claudin18.2-positive cancer cells by activating immune effector mechanisms (37). Patients with high claudin18.2 expression have received benefits on survival (36).To date, many studies have focused on expression and therapeutic usage of claudin18.2 in gastric cancer. Other organs have also been studied and some patients enrolled in clinical trials, but their number is limited (38). Therefore, whether they could benefit from the drug has not been validated. How claudin18.2 is expressed and associated with clinical features and survival is indicative of future treatment plans. To date, this is the first study to review and analyze the expression of claudin18.2 across human solid malignancies.

In all reviewed studies, 27.9% of patients showed expression of claudin18.2 in the primary resected tumor. Gastric cancer comprised the majority of the studies (13 out of 21, 61.9%) and 57.1% of patients (3,045 out of 5,331). Pooled analysis of clinicopathological features determined that claudin18.2 expression was linked to male predominance, T stage, positive MUC5AC, and EBV infection. Negative correlation with T stage was also recognized in subgroup analysis with gastric cancer. However, there were no significance statistical results between claudin18.2 expression and overall survival in gastric cancer. Previous studies also reported claudin18.2 overexpression in early gastric cancer, but survival outcomes did not show any correlation (28, 34, 39). This discrepancy might be due to various factors, such as nodal and metastatic stages, different subtypes of adenocarcinoma that affect prognosis, and patients who received adjuvant therapies. Similar results were observed in the studies of claudin18.2 expression in pancreatic cancer. The classic type of pancreatic cancer, which has a more favorable prognosis than the basal type, and precursor lesions frequently expressed claudin18.2 (40, 41), despite our study result showing no significant difference in T stage and survival of pancreatic cancer.

High claudin18.2 was correlated with positive MUC5AC, which is a marker of foveolar epithelium and play a key role in mucosal protection. This is mostly associated with the fact that claudin18.2 is strongly expressed in foveolar differentiated types of gastric cancer. Correlation between MUC5AC and claudin18.2 has been demonstrated in gastric cancer and colitis-associated colorectal adenocarcinoma (34, 42). On the other hand, decreased MUC5AC expression has been linked to carcinogenesis, but there is controversy among several studies and further research is needed (43). EBV-associated gastric cancer showed a higher incidence of claudin18.2 expression, which was formally documented in individual studies (44, 45). It was suggested that claudin18.2 may play a role in maintenance of EBV infection by mediating cell-to-cell contact (46). A previous meta-analysis of gastric cancer, which was limited to correlation between claudin18.2 and clinical features, did not show such significance between EBV and claudin18.2 expression (47). This is partially due to the fact that the previous study had fewer articles included for the meta-analysis, as well as one of the articles having patients who underwent neoadjuvant therapy (48).

No significance was observed between the expression of claudin18.2 and OS or DFS/TSS/PFS/RFS across all cancer types. However, in a subgroup analysis of lung cancer, the expression of claudin18.2 showed a significant association with improved OS. This suggests that claudin18.2 could serve as a potential novel biomarker related to prognosis in lung cancer. Notably, this favorable association was observed in the expression of epidermal growth factor receptor wild-type and low programmed cell death-ligand 1 expression despite the lack of statistical significance (29). In pancreatic cancer, claudin18.2 expression was marginally associated with a favorable OS (*p* = 0.058). However, gastric cancer did not exhibit significance in relation to claudin18.2 expression for both OS and DFS/TSS/PFS/RFS. In all included studies, no patient had received neoadjuvant therapy. Therefore, in pancreatic and gastric cancers, the improvement of survival in patients who have taken claudin18.2 inhibitors could be interpreted entirely as the effect of the drug. Consequently, these candidates would benefit from targeting claudin18.2, potentially leading to favorable survival outcomes.

There are some limitations to our study. First, most studies dealt with gastric cancer. Only two studies were available for prognostic features associated with lung cancer, with one study including ten times the number of participants as the other. Thus, caution is required when interpreting the subgroup analysis of lung cancer. Further studies should be conducted to establish its legitimacy. Second, many of the included studies indicate “claudin18.2”, but it is uncertain whether the employed antibodies actually distinguished between claudin18.1 and 18.2. Nevertheless, to the best of our knowledge, most carcinomas that express claudin18 have been identified as claudin18.2 (21–24, 26, 29–32, 34, 35). Lastly, the included studies adopted cut-off values for claudin18.2 positivity at their discretion, as no standard has been established. Future studies would benefit from the standardized protocol for assessing claudin18.2 positivity to ensure more reliable and comparable results.

In conclusion, claudin18.2 was expressed in various solid tumors and associated with diverse clinicopathological features as well as prognosis in lung cancer. Our investigation, along with recent advances in claudin18.2 target therapy, will serve as a foundation for future adoption in solid tumors, thereby expanding treatment options and enhancing patient survival.

## Data Availability

The original contributions presented in the study are included in the article/**Supplementary Material**. Further inquiries can be directed to the corresponding authors.
