# Supplementary material for: Clinicopathological significance and prognostic values of claudin18.2 expression in solid tumors: a systematic review and meta-analysis
Source: Front Oncol. 2024 Nov 20;14:1453906. doi: 10.3389/fonc.2024.1453906 (PMC11614718; doi:10.3389/fonc.2024.1453906)
Supplement: Supplementary file 1 [file DataSheet1.docx]

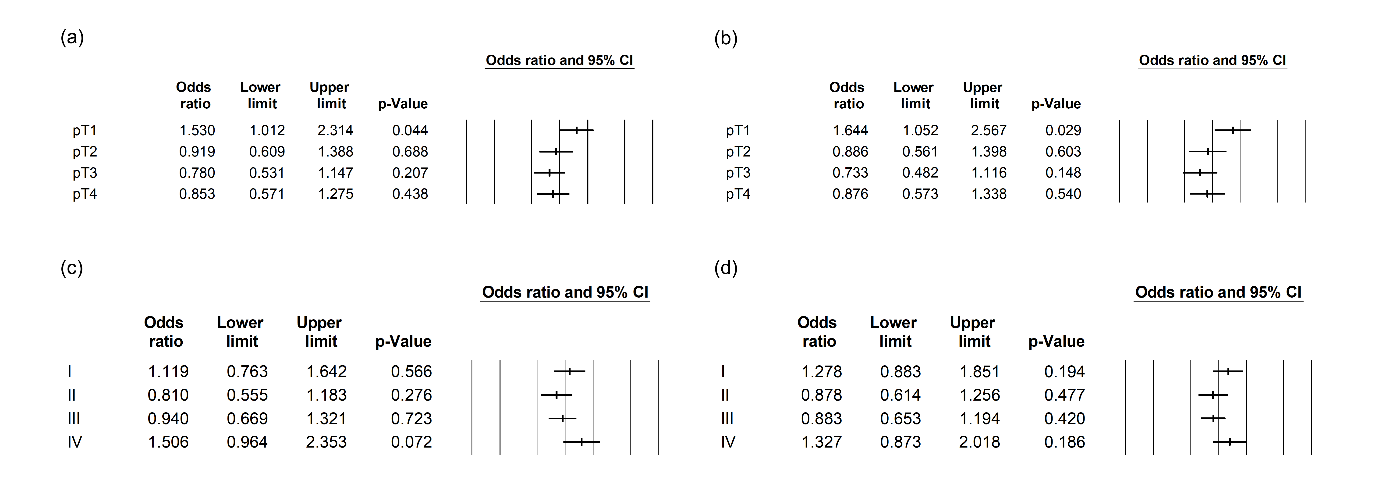


**Supplementary Figure 1** Forest plot describing the correlation between pT stage (a) and tumor stage (c). Subgroup analysis for pT stage and tumor stage in gastric cancer is shown in (b) and (d), respectively.
